# Supplementary material for: Epigenetic Marks, DNA Damage Markers, or Both? The Impact of Desiccation and Accelerated Aging on Nucleobase Modifications in Plant Genomic DNA
Source: Cells. 2022 May 25;11(11):1748. doi: 10.3390/cells11111748 (PMC9179523; doi:10.3390/cells11111748)
Supplement: Supplementary file 1 [file cells-11-01748-s001.zip › Table S1.pdf]

Table S1: Table of coordinates of variables used in principal component analysis.

Treatment	Dim.1	Dim.2	Dim.3	Dim.4	Dim.5
8-oxoG	0.57203	-0.70221	0.290405	-0.29132	-0.0405
hm^5C	-0.24924	-0.80787	-0.53045	0.01204	0.040057
m^5C	-0.95521	0.030831	0.052684	0.098291	-0.20248
ROS	0.764997	-0.47281	0.31759	0.27463	-0.01285
TAC	-0.93183	-0.01832	0.204887	-0.03055	0.279445
NPAC	-0.94239	-0.00741	0.091445	-0.2503	-0.08704
RG	-0.93477	-0.29882	0.085611	0.0937	-0.04589
TTC	-0.91495	-0.31488	0.146278	0.134775	0.01635
