# Supplementary material for: Epigenetic Marks, DNA Damage Markers, or Both? The Impact of Desiccation and Accelerated Aging on Nucleobase Modifications in Plant Genomic DNA
Source: Cells. 2022 May 25;11(11):1748. doi: 10.3390/cells11111748 (PMC9179523; doi:10.3390/cells11111748)
Supplement: Supplementary file 1 [file cells-11-01748-s001.zip › Table S2.pdf]

Table S2: Table of loadings matrix of variables used in principal component analysis.

Treatment	Dim.1	Dim.2	Dim.3	Dim.4	Dim.5
8-oxoG	6.111309	31.62745	15.37786	32.56347	1.238188
hm^{5}C	1.160185	41.86123	51.30706	0.055621	1.211469
m^{5}C	17.04093	0.060967	0.506117	3.706983	30.95564
ROS	10.92989	14.33857	18.3916	28.93924	0.124741
TAC	16.21679	0.021534	7.65452	0.358023	58.95858
NPAC	16.58673	0.003521	1.524794	24.03825	5.719585
RG	16.31951	5.727408	1.336421	3.368794	1.589961
TTC	15.63465	6.359318	3.901624	6.969616	0.201836
